# Supplementary material for: The interactome of intact mitochondria by cross-linking mass spectrometry provides evidence for coexisting respiratory supercomplexes
Source: Mol Cell Proteomics. 2017 Dec 8;17(2):216–32. doi: 10.1074/mcp.RA117.000470 (PMC5795388; doi:10.1074/mcp.RA117.000470)
Supplement: Supplemental Data [file supp_RA117.000470_133922_0_supp_23978_fzffwf.zip › spectra_annotation/mito_spectra_annotation/256-1-8-1-10-1.pdf]

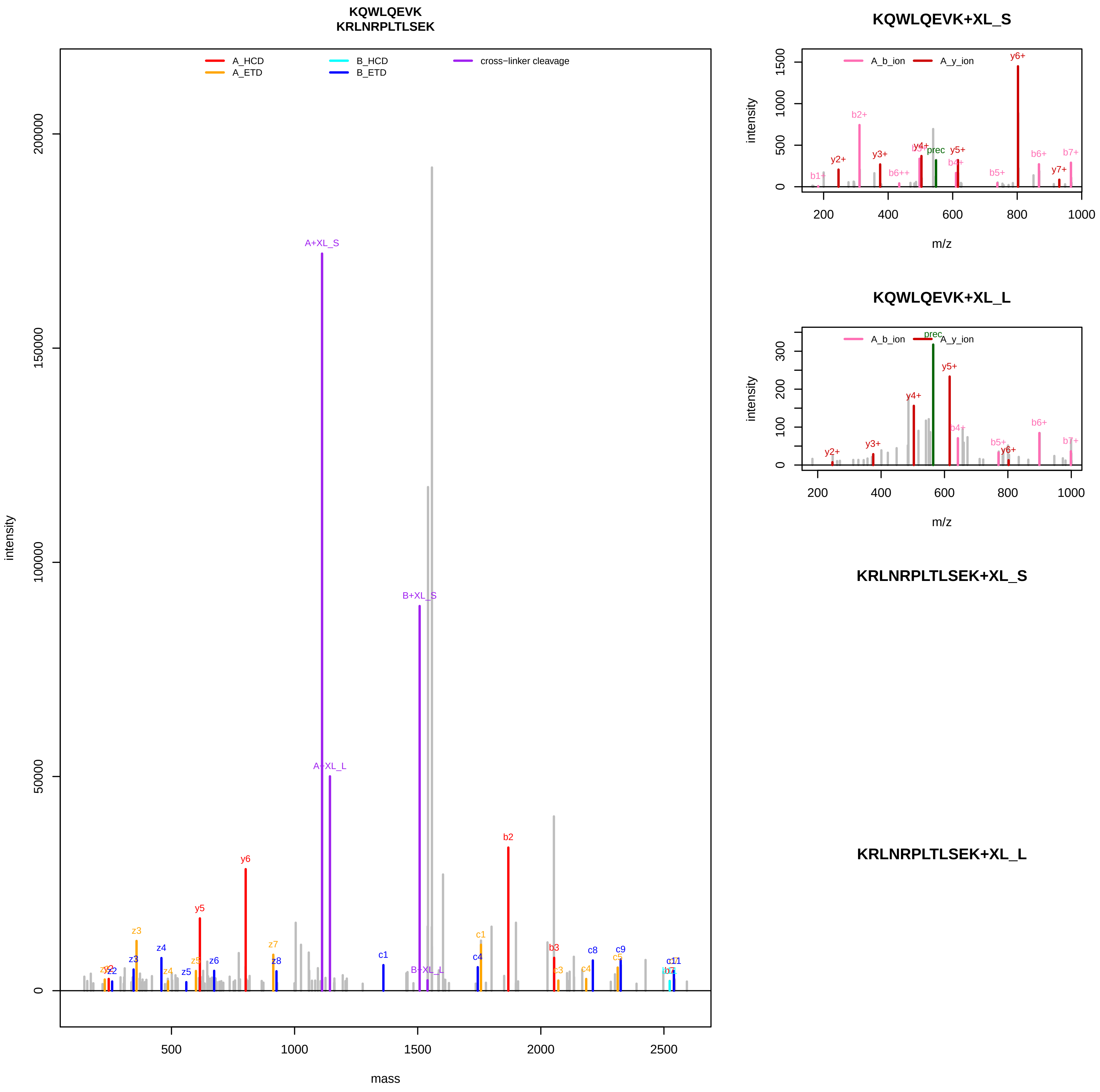
KQWLQEVK
KRLNRPLTLSEK
A_HCD
A_ETD
B_HCD
B_ETD
cross−linker cleavage
A+XL_S
B+XL_S
A+XL_L
B+XL_L
y5
y6
b2
b3
z3
z4
z5
z6
z7
z8
c1
c3
c4
c5
c8
c9
c11
intensity
mass
0
50000
100000
150000
200000
500
1000
1500
2000
2500
KQWLQEVK+XL_S
A_b_ion
A_y_ion
b1+
b2+
b6++
b4+
b5+
b6+
b7+
y2+
y3+
y4+
y5+
y6+
y7+
prec
m/z
KQWLQEVK+XL_L
KRLNRPLTLSEK+XL_S
KRLNRPLTLSEK+XL_L
